# Neurodevelopment in Children Exposed to Zika Virus: What Are the Consequences for Children Who Do Not Present with Microcephaly at Birth?

**DOI:** 10.3390/v13081427

**Published:** 2021-07-22

**Authors:** Paula Fabiana Sobral da Silva, Sophie Helena Eickmann, Ricardo Arraes de Alencar Ximenes, Celina Maria Turchi Martelli, Elizabeth B. Brickley, Marília C. Lima, Ulisses R. Montarroyos, Maria Durce Costa Gomes de Carvalho, Laura Cunha Rodrigues, Thalia Velho Barreto de Araújo, Liana O. Ventura, Danielle Maria da Silva Oliveira, Regina Coeli Ferreira Ramos, Demócrito de Barros Miranda-Filho, on behalf of the Microcephaly Epidemic Research Group (MERG)

**Affiliations:** 1 Pós-Graduação em Ciências da Saúde, Universidade de Pernambuco, Recife 50100-010, Brazil; raaximenes@uol.com.br (R.A.d.A.X.); ulisses.montarroyos@upe.br (U.R.M.); mdurce2@gmail.com (M.D.C.G.d.C.); dlsilva@gmail.com (D.M.d.S.O.); demofilho@gmail.com (D.d.B.M.-F.); 2 Departamento de Neurologia Infantil, Hospital Universitário Oswaldo Cruz, Recife 50100-130, Brazil; 3 Departamento de Pediatria, Universidade Federal de Pernambuco, Recife 50670-420, Brazil; sophie.eickmann@gmail.com (S.H.E.); limamc@gmail.com (M.C.L.); 4 Instituto Aggeu Magalhães—Fundação Oswaldo Cruz (Fiocruz), Recife 50740-465, Brazil; turchicm@gmail.com; 5 Department of Infectious Disease Epidemiology, London School of Hygiene & Tropical Medicine, London WC1E 7HT, UK; elizabeth.brickley@lshtm.ac.uk (E.B.B.); laura.rodrigues@lshtm.ac.uk (L.C.R.); 6 Departamento de Medicina Social, Universidade Federal de Pernambuco, Recife 50670-420, Brazil; thalia.araujo@ufpe.br; 7 Departamento de Oftalmologia, Fundação Altino Ventura, Recife 52171-011, Brazil; lianaventuramd@gmail.com; 8 Departamento de Infectologia Pediátrica, Hospital Universitário Oswaldo Cruz, Recife 50100-130, Brazil; coeliramos@hotmail.com

**Keywords:** child development, congenital Zika, Bayley scales, paediatric cohort, neuroclinical examination

## Abstract

The relation of Zika virus (ZIKV) with microcephaly is well established. However, knowledge is lacking on later developmental outcomes in children with evidence of maternal ZIKV infection during pregnancy born without microcephaly. The objective of this analysis is to investigate the impact of prenatal exposure to ZIKV on neuropsychomotor development in children without microcephaly. We evaluated 274 children including 235 ZIKV exposed and 39 controls using the Bayley-III Scales of Infant and Toddler Development (BSIDIII) and neurological examination. We observed a difference in cognition with a borderline p-value (*p* = 0.052): 9.4% of exposed children and none of the unexposed control group had mild to moderate delays. The prevalence of delays in the language and motor domains did not differ significantly between ZIKV-exposed and unexposed children (language: 12.3% versus 12.8%; motor: 4.7% versus 2.6%). Notably, neurological examination results were predictive of neurodevelopmental delays in the BSIDIII assessments for exposed children: 46.7% of children with abnormalities on clinical neurological examination presented with delay in contrast to 17.8% among exposed children without apparent neurological abnormalities (*p* = 0.001). Overall, our findings suggest that relative to their unexposed peers, ZIKV-exposed children without microcephaly are not at considerably increased risk of neurodevelopmental impairment in the first 42 months of life, although a small group of children demonstrated higher frequencies of cognitive delay. It is important to highlight that in the group of exposed children, an abnormal neuroclinical examination may be a predictor of developmental delay. The article contributes to practical guidance and advances our knowledge about congenital Zika.

## 1. Introduction

The link of Zika virus (ZIKV) to neurological impairment was described based on the outbreak of congenital anomalies observed in Brazil beginning in October 2015 [1,2,3]. The short-term risks of congenital ZIKV infections are already known and the impairment of neurodevelopment is evident in children with moderate to severe microcephaly [4,5,6]. However, the risk of developmental delays in children with evidence of maternal ZIKV infection during pregnancy born without microcephaly remains unknown [7,8,9].

Recognizing that congenital ZIKV infections may result in later-life consequences for children born ‘apparently asymptomatic’ for Congenital Zika Syndrome (CZS), the Microcephalic Epidemic Research Group (MERG) followed up a cohort of exposed children in order to identify abnormalities detected at later ages [10]. This paper reports on neurological and neurodevelopmental findings in children with no abnormal findings identified at birth, such as microcephaly, born to mothers who had evidence of ZIKV infection in pregnancy.

In a previously published study, the MERG conducted a neurodevelopmental assessment with a screening instrument and found a gradient of risk of developmental delay according to head circumference. Children with severe microcephaly were at highest risk for delays, while normocephalic ZIKV-exposed children had similar risks as control children [11]. With the current study, we sought to expand the assessment by using the Bayley-III Scales of Infant and Toddler Development (BSIDIII), which is a more specific instrument for evaluating cognitive, language, and motor development [12,13].

Since standardized instruments, such as the BSIDIII, are resource intensive and require lengthy evaluation times, specialized equipment investment, and trained professionals, they often have limited utility outside of a research setting. Therefore, the authors also examined complementary clinical markers that would correlate with the BSIDIII results and facilitate the identification and early referral of children at a higher risk of developmental delays for a more comprehensive assessment.

This investigation is part of a 5 year longitudinal study of the impacts of ZIKV infections during pregnancy and its main objective is to investigate the neuropsychomotor development of a group of children born in the state of Pernambuco, Brazil, with prenatal exposure to ZIKV without microcephaly.

## 2. Materials and Methods

### 2.1. Study Design

This investigation is a cross-sectional study including children followed-up as part of an ongoing prospective cohort (MERG-Paediatric Cohort) born between December 2015 and February 2019, during and after the peak of a microcephaly epidemic that occurred in northeastern Brazil [10].

### 2.2. Setting

The study was conducted at the Hospital Universitário Oswaldo Cruz, which is a reference hospital providing paediatric neurological care in Recife, Pernambuco, Brazil. The study was conducted by a multidisciplinary team, which included professionals trained in neurodevelopmental assessment. Before assessment, caregivers signed informed consent forms for their children’s participation. Ethical approval for follow-up in the MERG Paediatric Cohort was provided by a local institutional review board (CAAE: 52803316.8.0000.5192).

### 2.3. Participants

We studied 274 children who completed neurodevelopmental evaluation using the BSIDIII instrument. We compared outcomes between an exposed group of 235 children born without microcephaly to mothers with evidence of ZIKV infection during pregnancy (MERG Pregnant Women Cohort/MERG-PWC) and a control group of 39 children without microcephaly, other brain abnormalities detectable by transfontanellar ultrasonography (TF-US) at birth, or other major malformations typical of CZS who were initially recruited as controls in the MERG case-control study [14]. All controls tested negative by qRT-PCR for Zika virus and by capture-IgM ELISA for IgM antibodies and were born to mothers who tested negative for ZIKV at the time of delivery. Exposure to ZIKV during pregnancy was based on a combination of longitudinal data from molecular (qRT-PCR), serologic (IgM e IgG3), and plaque reduction neutralization tests (PRNT) for ZIKV. Maternal ZIKV exposure was categorized according to the degree of evidence of infection. Pregnant women were classified as positive, suspected, or negative. The positive group was divided into three subcategories (robust, moderate, and limited evidence) according to the level of diagnostic evidence. Robust evidence was defined by RT-PCR +, seroconversion, or at least two positive serologic tests (IgM or IgG3) and PRNT50; moderate evidence was defined by IgM or IgG3 + or Seroconversion by PRNT50; and limited evidence as PRNT50 + (in pregnancy or within 6 months post-pregnancy). Suspected cases were defined as PRNT50 titer ≥ 20 and <100 (in pregnancy or 1 month post-pregnancy) or a non-negative result (unspecified titter ≥ 20) in pregnancy or within 1 month post-pregnancy. Negative cases had negative nucleic acid test and negative serologic tests. In the present study we included children born to mothers classified as positive (82.1%) or suspected (17.9%). A total of 76 mothers out of 235 had a positive PCR. The others were classified according to the other laboratory criteria. All recruited pregnant women with rashes had been notified to the Health Secretariat of the State of Pernambuco [15,16].

### 2.4. Clinical Assessment

Participants were evaluated by different specialists of the MERG team by using standardized protocols. Paediatric neurologists examined children for the following: seizures, impairment of consciousness/behavior, localized motor deficits, altered tonus or muscular trophism, pyramidal signs, developmental milestones (e.g., motor and language), reflex reactions, behavior, and responses to visual and auditory stimuli. TF-US was performed within the first six months of life to assess presence of calcifications, tecidual hypoplasia or atrophy, corpus callosum agenesis or dysgenesis, and ventricular abnormalities. When there was a clinical indication, children had computed tomography (CT) or magnetic resonance (MRI) brain scan performed. Ophthalmological evaluation included specialized tests, such as RetCam (Natus Medical Inc., Pleasanton, CA, USA), eye structural evaluations, and visual acuity testing.

Maternal demographic data and medical histories were collected through caregiver interview. Neonatal and postnatal clinical characteristics were collected during hospitalization and outpatient follow-up by the research team as well as a review of medical records.

### 2.5. Neurodevelopment Assessment

Children were referred for neurodevelopmental assessment (BSIDIII) as they participated in regular evaluations with the specialists from the research group, prioritizing the chronological proximity of these children to annual periodic evaluations and the availability of the tests. We evaluated with Bayley 235/336 (69.9%) of the MERG-PWC and 39/46 (84.8%) of the controls. The evaluations took place at the Hospital Universitário Oswaldo Cruz between June 2017 and March 2020 and included children aged 6 to 42 months. For premature children, adjustments were made to their chronological age to reflect gestations of <40 weeks. For those children who underwent two Bayley assessments, we considered the ones at the oldest age in this analysis since we assumed that the results of neuropsychomotor assessment in older children would be more predictive of the long-term developmental patterns [17]. We also analyzed the results of the Bayley assessment in relation to the trimester of pregnancy in which the infection occurred. At the end of the assessment, all caregivers were informed of their child’s results and received guidance for home stimulation.

### 2.6. Data Management and Statistical Analysis

All children who met the inclusion criteria and completed the neurodevelopmental evaluation were included. Data were double entered into a secure ‘Shared Data Platform’ designed and protected by the Informatics Sector of the Aggeu Magalhães Institute/Fiocruz/Pernambuco. Analyses were performed using the Statistical Package for the Social Sciences (SPSS), version 15.

The analysis was conducted in two steps. In the first step, we compared the exposed children with the control group in relation to Bayley scores. In the second step, we investigated the association between the Bayley scores and the neuroclinical assessment, ophthalmologic summary, and imaging findings for the ZIKV-exposed group.

We used chi-squared tests to compare proportions of categorial variables and the Fisher’s exact test was used when it was appropriate. Student’s *t*-tests were used to compare means differences of continuous variables. The statistical significance level was considered to be 5% and all testing was two-tailed.

## 3. Results

Of the maternal and clinical-sociodemographic variables compared between the 235 exposed and 39 unexposed control children, only the level of maternal education and children’s age at assessment differed between the groups (Table 1). Mothers from the control group had a higher level of education and the children were approximately 4 months older at the time of the BSIDIII assessment.

We analyzed the cut-off point (<–1SD or score below 85 points) of three domains (cognitive, language, and motor development) as well as the balanced scores of the subtests (cognitive, receptive and expressive communication, and fine and gross motor). The frequency of delays in the cognition domain differed between the groups, with the percentage of delay equal to 9.4% (22/235) for the exposed children, while no child in the control group scored below the cut-off point (*p* = 0.052). This association remained even after adjusting for maternal education and children’s age at the Bayley assessment (crude OR = 5.64; adjusted OR = 5.95). There was no difference in the percentage of delay for language development (12.3% versus 12.8%, *p* = 0.933). There was an increase in delayed gross and fine motor function in exposed children, but this did not reach statistical significance for the composite and balanced scores. No child presented with evidence of severe delays (<–2SD or score below 70 points) in any domain (Table 2).

In relation to gestational age, 35/235 (14.9%) pregnant women had rashes in the first trimester, 67/235 (28.5%) in the second trimester, 131/235 (55.7%) presented rashes in the third trimester, and two (0.9%) were missing information. There was no association between the trimester of rash during pregnancy and the result of the Bayley assessment (*p* = 0.802).

Table 3 considered some clinical and imaging factors that could indicate a higher risk of neurodevelopmental abnormalities. Out of the exposed children with abnormalities on clinical neurological examination, 46.7% (14/30) also had BSIDIII abnormalities in any of the domains (Total Bayley Score). In contrast, the frequency of BSIDIII abnormalities was 17.8% (33/185) for children without neurological alterations (*p* = 0.001). Neither ophthalmologic nor radiologic abnormal findings were significantly associated with delay in any sub-area of the BSIDIII. The neurological alterations most often observed were altered muscle tone, irritability, behavioral complaints, and language delay observed clinically without the use of standardized screening instruments.

Abnormalities at neuroclinical examination were associated with delays in the language and motor domains (*p* = 0.003 and *p* = 0.008, respectively), but showed no association with cognitive development (Table 4).

## 4. Discussion

We present the results from neurodevelopmental evaluation of one of the largest samples ever studied relative to children exposed to ZIKV without microcephaly compared to a control group without prenatal ZIKV exposure. Using the BSIDIII cut-off point, we observed a slightly statistically significant difference for cognition, with mild to moderate delay in 9.4% of exposed children and 0.0% of the control group. Regarding language and motor, the percentage of delays did not differ between the exposed and the controls. In exposed children, the neuroclinical examination was predictive of neurodevelopment delay and showed significant association with the BSIDIII for the language and motor function domains.

Although the ZIKV epidemic in Brazil (2015–2016) has provided increasing scientific knowledge about CZS, later repercussions on neurodevelopment still require further observation and follow-up time to be completely understood. CZS is considered as a spectrum of manifestations, with severity related to the degree of microcephaly as well as other structural brain malformations [11,18,19]. Some authors have described the severe and early neurological impairment of children born with microcephaly, for whom more than 90% may present with neurodevelopmental delays [4,6,20]. Similar patterns have been observed in children born with normal head circumference who presented with decelerated growth rates and developed microcephaly within the first months of life [21].

The aim of this study was to evaluate children in a population unlike those previously mentioned. This study advances our knowledge by comparing children with evidence of maternal ZIKV infection during pregnancy but without microcephaly to a control group without evidence of maternal ZIKV infection during pregnancy born in the same region and period of time with similar sociodemographic characteristics.

Our overall results are consistent with another Brazilian study, which analyzed a smaller sample and reported a difference between groups concerning the cognitive domain but not for the motor and language domains [8]. Pursuing a more detailed comparison between groups, we analyzed the outcomes of the BSIDIII subtests using balanced scores for receptive and expressive communication as well as gross and fine Motor; the frequency of delays in these subtests did not differ significantly between the exposed and unexposed groups.

A survey conducted in Rio de Janeiro, Brazil, evaluated 146 ZIKV exposed children with the BSIDIII test and found that 28% presented with a mild to moderate delay in at least one domain of the composite BSIDIII score. Twelve percent of this sample scored below −2 SD (i.e., indicating severe delays) in at least one of the domains; the group with severe delays included six children with microcephaly and three who developed autism in the second year of life [22]. Another group of researchers evaluated a smaller sample of 26 children without microcephaly, but with other comorbidities such as macrocephaly and co-infections with HIV and toxoplasmosis and found the frequency of abnormal BSIDIII results in at least one domain to be 34.6% [23]. In our study, we did not observe severe impairment in any child and the percentage of delay for the three domains was less than those previously mentioned: at 9.4% for cognition, 12.3% for language, and 4.7% for motor development. It is likely that such difference resulted from the fact that we assessed a more homogeneous sample that excluded children with microcephaly or other severe disorders.

Another study applied BSIDIII to children exposed in utero to ZIKV paired with an external control group [24]. Developmental abnormalities were detected in 17.8% of the cases, while 100% of the controls scored above the cut-off point for delays. In that study, there was a substantial difference between the two groups, possibly because the control group was taken from an Austrian routine database. Meanwhile, our total sample was composed of Brazilian children from the same state who were predominantly from a disadvantaged social background.

The fact that this research took place in the state of Pernambuco, Brazil, which is the epicenter of the microcephaly epidemic, made it possible to evaluate a large and homogeneous sample. As for perinatal and sociodemographic factors, we detected only a slight increase in maternal education level for the control group. Another aspect differing between the groups is the mean age at the assessment time, which was 4 months older in the unexposed group compared to the exposed group. Although the literature frequently shows an impact of these factors on child development, in our study the slight association observed between in utero exposure to ZIKV and cognitive development remained even after adjusting for both maternal education and children’s age.

Since the present study is a cross-sectional study nested in a cohort, several aspects beyond neuropsychomotor development were assessed over time. When designing the study, we took into consideration the various clinical presentations of congenital ZIKV infection, which required detailed clinical evaluation essential to address the lack of knowledge about the disease at that time. Thus, the research group emphasized not only the application of the BSIDIII but also a detailed neurological assessment conducted with a standardized form, including a detailed history, physical examination, and developmental milestones. Our sample was also carefully followed up regarding radiological and ophthalmological aspects.

Among the exposed children, the neuroclinical examination was the only one of the three sets of variables tested (i.e., with the others being ophthalmologic and imaging) that showed significant association with the BSIDIII for the language and motor function domains. The lack of association with the cognitive domain may be due to the difficulty in assessing it in isolated form since cognition is predominantly verified by sensory-motor skills in the first years of life. Children only start to appropriate the body schema and elaborate mental representations of the surrounding environment after the third year of life.

Of the 215 exposed children, 30 (14%) had some abnormalities upon traditional clinical neurological assessment. Hypotonia, speech delay, irritability, sleep pattern changes, and inadequate visual response were the main complaints. Among the children with normal neurological examinations, 82% had an appropriate development, whereas in those with altered neurological examination only 53% had appropriate development. Therefore, abnormal neuroclinical examination indicated a markedly higher risk of neurodevelopmental delay in our study.

BSIDIII is considered a complex assessment. It is a difficult-to-apply and high-cost instrument that requires specific training and therefore is not available in most public services. Thus, we emphasize the importance of detailed clinical neurological examination as a tool to detect neurodevelopmental delays, which will enable the early identification and referral of children who require comprehensive assessment. The comprehensive neurological evaluation enables a better use of resources and the earliest possible intervention.

We found no association between ophthalmologic testing in the exposed group and Bayley outcome, which is different from findings among children with ZIKV-related microcephaly where a large percentage had severe visual impairment and neurodevelopmental delay. Of the 18 exposed children with altered ophthalmological examinations, 13 had a normal neurodevelopmental assessment (72.2%).

Except for serological tests and brain ultrasounds, additional tests were only requested as clinically indicated because of the ethical implications of the risk inherent to procedures under sedation in children. This explains the proportion of children without brain imaging and could account for the lack of association between neuroradiological findings and neurodevelopmental outcomes. Of the 16 children from the exposed group who exhibited altered brain ultrasounds (i.e., with calcifications, cerebellar hypoplasia and/or atrophy, and agenesis and/or dysgenesis of the corpus callosum and/or diffuse cortical atrophy), only two showed clinical changes which manifested as irritability in addition to developmental delay by the BSIDIII. Conversely, Moreira and colleagues identified a significant association between normal brain imaging and delays identified in the BSIDIII test [25].

Our study assessed a large and well-characterized sample of children born to women with laboratory evidence of ZIKV infection during pregnancy and without microcephaly who represented a relatively homogeneous and deprived sociodemographic background. These children were born at the epicenter of the ZIKV epidemic and represent a group that is still poorly studied in terms of neuropsychomotor development, especially because CZS is a novel disease for which the natural history is still being defined. Our comparison group was smaller, and this may have contributed to the lack of statistical significance of the observed increase in motor development delays. Another limitation is due to the fact that the children from the control group were born to mothers who were tested only at the time of delivery, rather than prospectively throughout pregnancy.

Overall, our findings suggest that relative to their unexposed peers, the ZIKV-exposed children without microcephaly are not at considerably increased risk of neurodevelopmental impairment in the first 42 months of life. However, a small group of children did demonstrate higher frequencies of cognitive delay, with a borderline significance. It is important to highlight that in the group of exposed children, an abnormal neuroclinical examination may be a predictor of developmental delay. However, it is also noteworthy that not all ZIKV-exposed children develop brain damage or suffer long-term neurodevelopmental impairment. These findings combined with the knowledge that children born in the epidemic are still at preschool age reinforces the importance of continued follow-up and detailed paediatric and neurological evaluation in the longer term, since there is still not enough knowledge about late-onset manifestations or more mild impairments that are not noticeable in the early years but exacerbated later, such as attention deficit hyperactivity disorder, autism, learning disability, and behavioral changes.

## Figures and Tables

**Table 1:** Selected characteristics of mothers and children with prenatal exposure to ZIKV born without microcephaly and the group of controls.

Variables	Total		Exposed		Controls		*p*-Value
	*n*= 274	%	*n* = 235	%	*n* = 39	%	
Maternal Race/Ethnicity (*n* = 273)							0.458
Mixed race	188	68.9	160	68.4	28	71.8	
White	51	18.7	46	19.7	5	12.8	
Black	31	11.4	26	11.1	5	12.8	
Other	3	1.1	2	0.9	1	2.6	
Maternal Years of Education (*n* = 273)							<0.001
0–8 years	98	35.9	87	37.1	11	28.2	
9–11 years	131	48.0	119	50.9	12	30.8	
>12 years	44	16.1	28	12.0	16	41.0	
Maternal Social Class (*n* = 262)							0.812
A, B1, B2	29	11.1	24	10.7	5	13.2	
C1	41	15.7	34	15.2	7	18.4	
C2	101	38.5	89	39.7	12	31.6	
D	91	34.7	77	34.4	14	36.8	
Prematurity (Gestational Age < 37 weeks)							1.00 ^a^
Yes	20	7.3	17	7.2	3	7.7	
No	254	92.7	218	92.8	36	92.3	
Children’s Birth Weight (*n* = 247)							0.185
<2500 g	23	9.3	21	10.0	2	5.3	
2500–2999 g	61	24.7	55	26.3	6	15.8	
>3000 g	163	66.0	133	63.7	30	78.9	
Children’s Sex							0.674
Female	139	50.7	118	50.2	21	50.8	
Male	135	49.3	117	49.8	18	46.2	
Children’s Age at Bayley Assessment (Months) Mean (SD)	274	28.21 (7.35)	235	27.42 (7.35)	39	31.59 (6.32)	0.001 ^a^

^a^ Fisher’s exact test.

**Table 2:** Neurodevelopment of the group of children with prenatal exposure to ZIKV born without microcephaly and the group of controls based on BSIDIII composite and balanced scores.

BSIDIII	Domains	Test Performance	Exposed *n* = 235		Controls *n* = 39		*p*-Value
			*n*	% (CI 95%)	*n*	% (CI 95%)	
Composite Scores	Cognitive	Adequate	213	90.6	39	100	0.052 ^a^
		Mild to moderate delay	22	9.4 (6.3–13.8)	0	0	
	Language	Adequate	206	87.7	34	87.2	1.000 ^a^
		Mild to moderate delay	29	12.3 (8.7–17.2)	5	12.8 (5.6–26.7)	
	Motor	Adequate	224	95.3	38	97.4	1.000 ^a^
		Mild to moderate delay	11	4.7 (2.6–8.2)	1	2.6 (0.5–13.2)	
Balanced Scores	Expressive Language	Adequate	205	87.2	31	79.5	0.195
		Mild to moderate delay	30	12.8 (9.1–17.6)	8	20.5 (10.8–35.5)	
	Receptive Language	Adequate	218	92.8	36	92.3	1.000 ^a^
		Mild to moderate delay	17	7.2 (4.6–11.3)	3	7.7 (2.6–20.3)	
	Gross Motor	Adequate	223	94.9	38	97.4	0.701 ^a^
		Mild to moderate delay	12	5.1 (2.9–8.7)	1	2.6 (0.5–13.2)	
	Fine Motor	Adequate	229	97.4	39	100	0.599 ^a^
		Mild to moderate delay	6	2.6 (1.2–5.5)	0	0	

^a^ Fisher’s exact test.

**Table 3:** Clinical and radiological association with performance on the BSID III test in the children with prenatal exposure to ZIKV born without microcephaly.

Clinical and Radiological Assessment		Total Bayley Score						
		Total		Adequate Development		Delayed Development		*p*-Value
		*n*	(%)	*n*	(%)	*n*	(%)	
Neuroclinical Examination (*n* = 215)	Normal	185	86.1	152	(82.2)	33	(17.8)	0.001
	Altered	30	13.9	16	(53.3)	14	(46.7)	
Ophthalmologic findings (*n* = 172)	Normal	154	89.5	113	(73.4)	41	(26.6)	1.000 ^a^
	Altered	18	14.5	13	(72.2)	5	(27.8)	
Imaging findings (*n* = 142)	Normal	126	88.7	98	(77.8)	28	(22.2)	0.529 ^a^
	Altered	16	11.3	11	(68.7)	5	(31.3)	

^a^ Fisher’s exact test.

**Table 4:** Association between Bayley’s test and neuroclinical examination in the children with prenatal exposure to ZIKV born without microcephaly.

Neuroclinical Examination	Bayley III Subscales				*p*-Value
	Adequate Development		Delayed Development		
	*n*	(%)	*n*	(%)	
	Cognition				0.105 ^a^
Normal	171	(92.4)	14	(7.6)	
Altered	25	(83.3)	5	(16.7)	
	Language				0.003 ^a^
Normal	169	(91.4)	16	(8.6)	
Altered	21	(70.0)	9	(30.0)	
	Motor				0.008
Normal	182	(98.4)	3	(1.6)	
Altered	26	(86.7)	4	(13.3)	

^a^ Fisher’s exact test.

## Data Availability

Data cannot be shared. Public availability of the data would compromise patient privacy. De-identified data can be made available upon reasonable request from qualified investigators by contacting the Programa de Pós-Graduação em Ciências da Saúde (PPGCS) da Universidade de Pernambuco (UPE) at ppg.cienciasdasaude@upe.br.
